# Decision-Making Performance and Block-Wise Trajectories in Gambling Disorder and Opioid Use Disorder Using the Iowa Gambling Task

**DOI:** 10.3390/brainsci16101077

**Published:** 2026-10-08

**Authors:** Edward Jacek Gorzelańczyk, Aleksandra Kawala-Sterniuk, Ewa Laskowska, Piotr Kamiński, Piotr Walecki

**Affiliations:** 1 Department of Epistemology and Cognitive Science, Faculty of Philosophy, Kazimierz Wielki University, 85-092 Bydgoszcz, Poland; 2 Department of Medical Biology and Biochemistry, Faculty of Medicine, Collegium Medicum in Bydgoszcz, Nicolaus Copernicus University in Toruń, 85-092 Bydgoszcz, Poland; piotr.kaminski@cm.umk.pl; 3 Greater Poland Neuropsychiatric Center Named After Oskar Bielawski, Independent Public Healthcare Institution, 64-000 Kościan, Poland; 4 The Society for the Substitution Treatment of Addiction “Medically Assisted Recovery”, 85-791 Bydgoszcz, Poland; 5 Department of Artificial Intelligence, Faculty of Information and Communication Technology, Wrocław University of Science and Technology, 50-370 Wrocław, Poland; aleksandra.kawala-sterniuk@pwr.edu.pl; 6 Department of Cardiology and Internal Medicine, Faculty of Medicine, Collegium Medicum in Bydgoszcz, Nicolaus Copernicus University in Toruń, 85-094 Bydgoszcz, Poland; 7 Department of Nature Conservation, Institute of Biological Sciences, University of Zielona Góra, 65-516 Zielona Góra, Poland; 8 Department of Bioinformatics and Telemedicine, Faculty of Medicine, Jagiellonian University Medical College, 30-688 Kraków, Poland; piotr.walecki@uj.edu.pl

**Keywords:** Iowa Gambling Task, gambling disorder, opioid use disorder, methadone, decision-making, reward processing, reinforcement learning, executive control

## Abstract

Background/Objectives: Decision-making differences have been reported in substance-related and behavioral addictions, but Iowa Gambling Task (IGT) performance patterns may differ. We compared IGT performance in gambling disorder, opioid use disorder (OUD) before and approximately 90 min after methadone dosing, and healthy controls. Methods: The study enrolled 103 adult men: 33 with gambling disorder, 30 with opioid use disorder (OUD) receiving methadone maintenance, and 40 healthy controls. One control participant discontinued the IGT before completion, leaving 39 complete control outcome records. Between-cohort inference therefore used 39 controls, 33 participants with gambling disorder, and 30 OUD pre-methadone observations; pre-/post-dose inference used 27 complete OUD pairs as repeated measurements. Results: Net Total differed across the three independent baseline cohorts, F(2,99) = 8.73, *p* < 0.001, and η^2^ = 0.150. Controls had higher scores than the gambling-disorder cohort (Holm-adjusted *p* = 0.032) and the OUD pre-methadone cohort (*p* < 0.001); gambling disorder versus OUD pre-methadone was not significant (*p* = 0.083). In OUD, the within-subject condition effect was not significant, F(1,26) = 1.55, *p* = 0.224. Block-wise profiles are presented descriptively; gambling disorder showed a 2.79-point Block 3-to-4 decrease. Conclusions: The recruited male gambling-disorder and OUD baseline cohorts showed lower mean IGT performance than healthy controls. Block-wise trajectories differed descriptively; the late decline in gambling disorder is exploratory and requires replication with prespecified contrasts or mixed-effects models.

## 1. Introduction

A growing body of work frames addiction as a condition that strains the conventional notion of free will without eliminating agency, situating decision-making variability at the intersection of neurobiology and philosophy [1,2,3]. Philosophical accounts—especially compatibilist perspectives articulated by Levy [4] and Pickard [5]—distinguish diminished autonomy from absolute lack of freedom, arguing that emotional dysregulation and impaired self-control in addiction compromise, but do not abolish, the capacity for future-oriented choice [5,6]. Empirical and conceptual work further shows that top–downtop–down regulatory influence is weakened in addiction, producing vulnerabilities in goal-directed behavior while leaving room for responsibility and intervention [2,7,8]. This tension between disease-model descriptions and preserved aspects of agency highlights why tasks like the Iowa Gambling Task (IGT) are valuable: they can quantify how addictive processes bias reward evaluation and impulse control without reducing individuals to passive subjects [5]. Understanding these nuances is essential when comparing decision-making variability across gambling and OUD, as both involve altered valuation systems operating within a constrained but existing motivational architecture [9,10]. Gambling disorder has also been linked to increased impulsivity and context-dependent risk-taking, particularly in environments associated with reward cues such as sports betting [11]. Attitudes, beliefs, and impulsivity play an important role in the development and maintenance of online gambling disorder [12].

The IGT has been utilized in addiction research to evaluate decision-making strategies involving risk assessment and long-term planning. Studies have demonstrated that individuals with substance use disorders, such as alcohol and OUD, exhibit impaired IGT performance, indicating deficits in decision-making processes [13,14,15,16]. Addictive disorders, both substance-related (e.g., opioid use disorder) and behavioral (e.g., gambling disorder), share common cognitive and neurobiological characteristics, including impairments in impulse control and reward sensitivity [17,18,19,20,21,22]. Accumulating evidence indicates that such impairments are underpinned by functional abnormalities within the prefrontal cortex (PFC) and reward-related neural circuitry, including mesolimbic pathways, which bias decision-making toward immediate, high-magnitude rewards despite long-term negative consequences [23]. Recent findings link maladaptive risk-taking to disrupted prefrontal control and altered reward valuation across psychiatric and addictive disorders [24,25], highlight the role of impulsivity-related neurobiological and genetic factors [21], and show that steep delay discounting is a robust transdiagnostic marker [26]. Evidence from studies on pain and substance use disorders further implicates opioidergic and mesolimbic dysfunction in these decision-making biases [27], while meta-analytic data confirm widespread motivational network alterations [28]. Decision-making impairments are central to understanding both substance-related and behavioral addictions. The IGT has been extensively employed to characterize impairments in decision-making, providing a window into the underlying cognitive mechanisms of risk-taking behavior. It is used both in translational frameworks and clinical populations to dissociate components such as reward evaluation, learning under uncertainty, executive control, and stress-modulated choice behavior [29,30,31,32,33]. Empirical evidence indicates that individuals with cocaine dependence exhibit impaired prefrontal cortex function during decision-making, with disrupted activity and connectivity undermining future-oriented evaluation and impulse control. Cocaine-related reductions in gray matter and functional abnormalities in dorsolateral and orbitofrontal prefrontal regions have been linked to heightened impulsivity and maladaptive risk preferences [34,35,36,37,38].

Moreover, patterns of prefrontal engagement (including middle frontal gyrus activity) predict relapse risk, underscoring the region’s role in maintaining goal-directed, future-focused behavior [39]. Deficiencies in decision-making have been found in individuals with OUD. Previous studies indicate that individuals with OUD receiving methadone maintenance may perform worse on the IGT than healthy controls. Relative to healthy individuals, the poorer performance of persons with psychoactive substance dependence on the IGT reflects pronounced cognitive impairments, particularly within executive function domains and higher-order decision-making processes [40,41]. Substance use history independently predicts disadvantageous IGT performance even after controlling for general executive ability, indicating that these decision-making deficits are robust in populations with addiction [23,42]. Computational and empirical work further demonstrates altered preference dynamics and learning mechanisms in individuals with substance use disorders on the IGT, consistent with disrupted executive control and reward evaluation [43]. Clinical evidence suggests that short-term opioid-agonist treatment does not fully normalize neurocognitive performance, underscoring the persistence of decision-making and executive deficits [44,45,46]. Studies using computational modeling confirm that decision-making deficits persist in individuals with a history of opioid use, even after prolonged drug-free periods [47]. Moreover, neurorehabilitation research highlights that cognitive recovery in opioid and stimulant users is limited, emphasizing the need for targeted interventions to address long-standing deficits in executive function and decision-making [48].

Personality dimensions (e.g., high impulsivity, low conscientiousness) and affective dysregulation (including anxiety, depression, and emotional instability) in opioid use disorder substantially contribute to impaired decision-making by disrupting executive control and biasing reward valuation [48]. Emotion regulation difficulties and negative affective states amplify maladaptive choices, while trait-level factors moderate the extent of decision deficits [49]. High levels of trait and state anxiety, along with low self-directedness, have been associated with poorer IGT performance in this population. These findings highlight the complex interplay between emotional states and cognitive functions in addiction [49,50]. Gambling disorder, classified as a behavioral addiction, is likewise characterized by decision-making impairments. Affected individuals often prefer immediate rewards despite potential long-term negative consequences, reflecting deficits in impulse control and future-oriented thinking [51,52,53,54,55]. Functional neuroimaging studies suggest that both individuals with gambling disorder and those with substance dependence exhibit altered prefrontal engagement during decision-making tasks, consistent with partially shared neural substrates of these impairments [15,56,57,58,59]. Building upon previous explorations of subcortical loops, which involve intricate neural circuits connecting subcortical structures such as the basal ganglia with cortical regions, these circuits have been implicated in addiction and decision-making [14,23,60,61,62,63,64,65]. These loops are pivotal in modulating reward- and habit-related behaviors [66,67,68,69,70,71]. The PFC plays a crucial role in governing executive functions such as self-control, salience attribution, and awareness [72].

Recent neurobiological evidence indicates that addiction is associated with disrupted top–down control from the PFC over limbic reward circuitry (including the nucleus accumbens), leading to maladaptive valuation, impaired executive regulation, and a bias toward immediate reward-seeking despite negative consequences. This dysregulation undermines decision-making and increases susceptibility to compulsive addictive behaviors [25,56,73,74,75]. The reward system, particularly the mesocorticolimbic dopamine pathway, plays a central role in reinforcing behaviors by associating them with pleasurable outcomes [75]. Repeated exposure to addictive substances can dysregulate reward-related signaling and reinforce substance-seeking behavior [76]. Dysregulation within the reward system disrupts the balance between the prefrontal cortex and subcortical structures, leading to diminished executive control and increased impulsivity [68,75,77,78]. The interplay between subcortical loops and the PFC provides a neurobiological framework for understanding compromised decision-making in addiction [79]. Clarifying the relationships between cortical and subcortical structures within cortico-subcortical loops is important for developing targeted interventions aimed at restoring balance within these neural circuits, thereby improving treatment outcomes for individuals with addiction [80,81]. The cortico-subcortical loops, encompassing prefrontal-striatal and prefrontal–limbic connections, are crucial for integrating cognitive control with reward processing, thereby influencing impulsivity, risk-taking, and adaptive decision-making [63]. Disruptions in cortico-subcortical loops, particularly within the PFC and its connectivity with reward-related neural pathways, have been implicated in various forms of addiction [56,70,82]. In individuals with OUD, disruptions in cortico-subcortical neural circuits are associated with weakened inhibition of subcortical structures by the prefrontal cortex, leading to compulsive substance-seeking behavior despite negative consequences [83]. Neuroimaging studies have reported reduced activity in the ventromedial prefrontal cortex (vmPFC) and anterior cingulate cortex (ACC) in individuals with OUD relative to healthy controls; these regions contribute to reward evaluation and cognitive control over impulsive decision-making [84,85]. The medial prefrontal cortex plays a central role in integrating value signals and guiding goal-directed decision-making [86].

Chronic opioid use is associated with disrupted dopaminergic signaling to the nucleus accumbens, resulting in maladaptive reinforcement learning that prioritizes immediate rewards over long-term benefits [56,74,87,88]. In individuals diagnosed with gambling disorder, classified as a behavioral addiction, neurobiological similarities to substance-related disorders have been observed—particularly in the functioning of the PFC and reward-related structures connected to this system [89,90,91]. In individuals with gambling disorder, increased striatal activity and reduced functional connectivity between the dorsolateral prefrontal cortex (dlPFC) and limbic structures have been observed compared to healthy controls, a pattern that has been interpreted in relation to impulsivity and impaired decision-making under uncertainty [92]. In OUD, a blunted dopaminergic response is observed as a result of chronic substance exposure, whereas in gambling disorder, increased dopamine release in response to gambling-related cues is often noted, reinforcing the tendency toward risk-taking behavior [74,93,94]. Converging evidence links impaired decision-making processes with the development or persistence of addictive behaviors [56,95,96,97,98,99,100]. Clinical studies suggest that individuals with gambling disorder—particularly those engaged in non-strategic forms of gambling—exhibit greater cognitive deficits and emotional dysregulation compared to those engaged in strategic forms of gaming [101]. Gambling disorder is strongly associated with difficulties in decision-making processes, as assessed by tasks such as the IGT [102]. PET studies indicate that, in individuals with gambling disorder, striatal dopamine release peaks under conditions of maximum uncertainty rather than maximum reward, suggesting that their dopaminergic system reinforces behaviors driven by unpredictability [94,103,104]. Neuroimaging findings using the [123I]-FP-CIT SPECT technique indicate the presence of presynaptic dopaminergic dysfunction in the striatum of individuals with gambling disorder [105]. The presynaptic deficit in individuals with gambling disorder was correlated with impaired cognitive flexibility and attentional control, further supporting the role of dopaminergic dysregulation in the cognitive dysfunctions observed in addictive behaviors [106,107,108]. A key distinction between OUD and gambling disorder lies in how pharmacological interventions modulate risk-taking behavior [23]. Significantly reduced risky decision-making following methadone administration has been observed in individuals with OUD, as assessed by the IGT [23]. In contrast, no comparable pharmacologically induced changes have been identified in individuals with gambling disorder, suggesting that dopaminergic regulation of risk behavior may differ between these conditions. These findings suggest that, although both addictions engage the brain’s reward circuitry, their neurochemical mechanisms and responsiveness to pharmacological treatment diverge in important ways [23].

Methadone—a long-acting opioid agonist used in maintenance treatment—may support aspects of cognitive control by stabilizing the neurobiological state associated with OUD and modulating prefrontal-striatal function [14]. IGT studies suggest that individuals with OUD who have received methadone make more considered decisions than those who have not received an opioid agonist. However, their behavior remains riskier than healthy individuals in the control group [23,45,109]. Compared with individuals with OUD, those with gambling disorder may exhibit a distinct decision-making profile [23]. In individuals with gambling disorder, the tendency to engage in risky decision-making is not reduced by pharmacotherapy, but is shaped by emotional and cognitive responses to loss (punishment) and reward expectancy [23]. Healthy individuals, in comparison to those with gambling disorder, demonstrate greater cognitive flexibility, which enables them to learn from negative experiences and dynamically adjust their decision-making strategies [53,110]. The adjustment of response strategies to punitive and rewarding stimuli is regulated by proper functioning of the PFC, which enables the integration of risk assessment, impulse control, and long-term reward evaluation [21,45,72,111]. Subthalamic circuitry has also been implicated in the control of impulsive actions and risk evaluation [112]. The distinct decision-making profile observed in individuals with gambling disorder relative to those with substance-related addictions supports the need to consider disorder-specific neurocognitive deficits when developing therapeutic interventions [68,90,106,113]. The interaction between subcortical structures—particularly the striatum—and the PFC provides an important framework for interpreting impaired decision-making in addiction [56,79]. Differences in cortico-subcortical loop function across psychiatric and addictive disorders may therefore be relevant to the development of targeted interventions aimed at improving neural regulation and treatment outcomes [63,81,114,115]. Decision-making processes rely on complex cortical–subcortical interactions involving prefrontal and striatal circuits [116]. Computational models suggest that decision-making deficits may arise from alterations in excitation–inhibition balance within cortical circuits [117].

A comparison of cognitive-emotional decision-making deficits among individuals with different types of addiction reveals both shared features and characteristics specific to each addiction type [98,118,119,120]. Both individuals with OUD and those with gambling disorder exhibit impairments in performing the IGT, with working memory deficits being more pronounced in individuals with OUD [121,122]. These differences in decision-making strategies between individuals with gambling disorder and those with substance addiction, stemming from the distinct functioning of cortico-subcortical loops, may serve as a foundation for developing personalized intervention strategies targeting specific cognitive deficits associated with each type of addiction [23,113,123,124]. Clarifying the neurocognitive foundations of decision-making within cortico-subcortical loop models is important for developing practical treatment strategies [114,125,126,127]. It is advisable to implement therapeutic strategies that strengthen cognitive control and target the regulation of affective disturbances, thereby increasing the likelihood of reducing maladaptive decision-making patterns commonly observed in individuals with addiction [128,129]. This study builds upon prior research by investigating decision-making performance across three groups: (1) individuals with OUD assessed before and after methadone administration [127,130], (2) individuals with gambling disorder [23], and (3) a healthy control group [14]. By analyzing overall IGT performance and block-wise choice trajectories [23], this research aims to delineate the decision-making profiles associated with OUD and gambling disorder, as well as differences between the two clinically defined methadone-assessment sessions, interpreted in the context of existing cortico-subcortical circuit models [14,23,64].

The present work is a secondary analysis and analytical extension of our earlier investigation [23], rather than an independent replication. The 33-participant male gambling-disorder cohort and its IGT observations are the previously characterized cohort and were reanalyzed here within a revised comparison framework. The pre-dose OUD cohort was drawn predominantly from the same long-term clinical research programme and therefore substantially overlaps with the earlier dataset, while the control cohort was selected from the broader research population and is only partially overlapping. The present analysis uses a male-only comparison framework to reduce heterogeneity in sex composition, separates the three independent baseline cohorts from the repeated OUD observations, and examines pre-/post-dose performance within the same OUD participants. This design does not remove age-, recruitment-setting-, or other cohort-level differences, and the findings should not be interpreted as an independent replication of the earlier study.

A separate cohort meeting criteria for both gambling disorder and OUD was not included; therefore, the present study cannot estimate the combined effect of these diagnoses on IGT performance. Likewise, prefrontal function was not measured directly, and any discussion of PFC circuitry is grounded in external studies rather than a neural assessment of the present participants.

## 2. Materials and Methods

A total of 103 adult men were enrolled and divided into three groups: (1) individuals with opioid use disorder (OUD; *n* = 30); (2) individuals with gambling disorder (*n* = 33); and (3) healthy individuals from the control (comparison) group (*n* = 40). Recruitment targets were defined before analysis on the basis of statistical power considerations, with approximately 30 participants planned for each clinical cohort and 40 controls. Individuals with OUD (ICD-10: F11.2) were recruited from certified substitution-treatment programmes in Bydgoszcz and Gdańsk (Table 1), and individuals with gambling disorder (F63.0, ICD-10) were recruited at Bydgoszcz-Fordon Prison. Recruitment followed a two-stage procedure: the collaborating institution identified potentially eligible volunteers, after which study eligibility and the clinical diagnosis were independently confirmed for research purposes by a psychiatrist specializing in addiction treatment. The institutional referral did not itself constitute study qualification.

The control group consisted of healthy volunteers recruited through academic institutions (not exclusively university students), including the Jagiellonian University Collegium Medicum in Kraków and the Collegium Medicum of Nicolaus Copernicus University in Bydgoszcz. Controls were required to have no psychiatric disorder or addiction, with eligibility assessed clinically by qualified medical and psychological personnel, including an experienced psychiatrist specializing in addiction medicine. Only individuals aged 21–60 years were included. Exclusion criteria comprised a history of psychotic disorders, head injuries, current intoxication with psychoactive substances (alcohol or drugs), withdrawal symptoms, and cognitive impairment. Participation was voluntary in all recruitment settings. Potential participants were informed in advance, received no financial remuneration or institutional benefit for participation, and could refuse or discontinue the study at any time without giving a reason and without penalty, effect on treatment or substitution care, or effect on custodial status or institutional privileges. The cohorts were not individually matched for age or recruitment setting; these variables are therefore considered potential confounders in interpretation of between-cohort differences.

The study was conducted in accordance with the Declaration of Helsinki under the research protocol approved by the Bioethics Committee of Collegium Medicum in Bydgoszcz, Nicolaus Copernicus University in Toruń, Poland (original approval No. KB 416/2008; continuation and extension approved on 14 September 2021, with authorization to continue the research through 31 December 2027). Written informed consent was obtained from all participants before study procedures.

**Figure 1:**
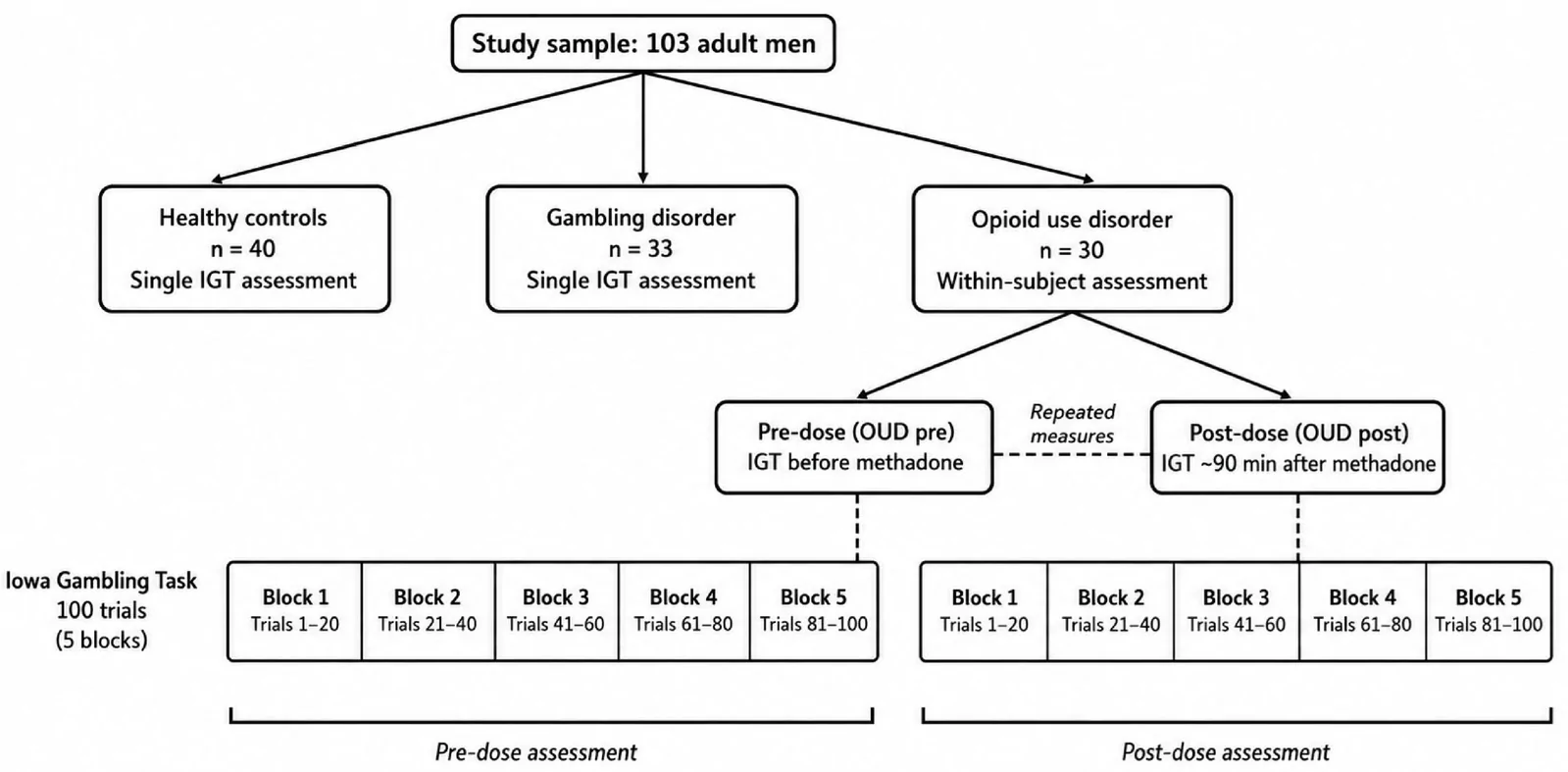
Study design and analysis denominators. The study enrolled 103 adult men. Thirty-three participants with gambling disorder completed a single IGT assessment; 40 healthy controls were enrolled and 39 completed the IGT; and 30 participants with OUD completed the pre-dose assessment and 27 completed both pre- and approximately 90-min post-dose assessments. Paired OUD inference used only the 27 complete pairs.

Cognitive performance was assessed using the Mini-Mental State Examination (MMSE); the inclusion criterion was a minimum MMSE score of 27 points. Individuals with OUD were receiving clinically stable methadone substitution treatment for at least two weeks before the study (mean daily methadone dose: 70.4 ± 11.9 mg). Methadone administration was routine clinical care and was not an experimental intervention introduced by the research protocol; dose, indication, and treatment schedule were determined independently by the substitution-treatment programme. The IGT was administered twice to the same OUD participants: (1) before the scheduled daily methadone dose (OUD pre-methadone) and (2) approximately 90 min after routine dosing (OUD post-methadone) (Figure 1). All 30 participants completed the pre-dose assessment. Three did not complete the second assessment; participation in the second session remained voluntary and no reason for non-participation was required. Accordingly, 27 participants contributed complete paired pre-/post-dose observations. Valid pre-dose observations from all 30 participants were retained in the independent baseline comparison, whereas paired inference was restricted to the 27 complete pairs. No post-dose values were imputed. The pre-dose assessment occurred during ongoing maintenance treatment and did not represent an opioid-free baseline.

The approximately 90-min post-dose assessment interval was chosen deliberately to capture the early period in which patients commonly reported pronounced subjective psychological effects following the daily dose, not the later expected maximum plasma concentration. The protocol was informed by previous investigations from our group [14,127,131] and preliminary, unpublished clinical observations in selected patients who completed IGT assessments at approximately hourly intervals over five hours. In these preliminary observations, behavioral changes of interest occurred around 1–2 h after dosing, whereas later assessment could be complicated by reduced attention and difficulty completing the task. The research protocol did not alter the timing or dose of routine methadone administration. The fixed test sequence therefore contrasts two clinically defined assessment sessions rather than isolating an acute pharmacological effect.

Risk-taking behavior was assessed using the computerized Iowa Gambling Task (IGT) implemented in Psychology Experiment Building Language (PEBL), version 2.1 [132]. The task consisted of 100 trials divided into five 20-trial blocks. Four virtual decks (A, B, C, and D) were presented; A and B are conventionally disadvantageous and C and D advantageous. The primary outcome was the conventional IGT net score, calculated for each block and for the complete task as the number of selections from advantageous decks (C + D) minus selections from disadvantageous decks (A + B). Raw PEBL trial files were subsequently processed with in-house batch-processing software. For the analyses reported here, Net Total and block-wise net scores were derived from deck selections using the conventional IGT scoring rule. The original trial-level PEBL records and the processed analytical data were retained for verification and reproducibility.

Statistical analyses were structured to respect the dependence of repeated OUD observations and the analysis-specific denominators. For the primary between-cohort comparison of Net Total, the analysis included 39 healthy controls with complete IGT outcomes, 33 participants with gambling disorder, and 30 OUD pre-methadone observations. A one-way ANOVA assessed the overall cohort effect, with eta squared (η^2^) reported as the effect size. Pairwise contrasts were evaluated using Welch *t* tests with Holm adjustment for three comparisons, and Hedges g was reported as a standardized effect size. The OUD pre- versus post-methadone condition effect was analyzed within subjects in the 27 participants with complete paired observations; the three OUD participants who did not complete the second assessment were not included in paired inference. Because the condition factor has two levels, its main effect does not require a sphericity assumption. In the revised analysis, block-wise pre-/post-dose profiles are presented descriptively and are not used for confirmatory block or condition-by-block inference. No missing outcome values were imputed.

Block-wise trajectories were treated as secondary descriptive analyses. The Block 4 decrease in the gambling cohort was therefore interpreted as an exploratory temporal pattern rather than a validated diagnostic marker or a confirmed learning mechanism. Correlations between Net Total and the advantageous/disadvantageous component counts were not retained because these variables are algebraically coupled by the definition of the IGT net score and do not represent independent empirical associations. Net Total remained the primary outcome.

## 3. Results

### 3.1. Overall IGT Performance Across Independent Cohorts

Among participants with complete baseline IGT outcomes, healthy controls (*n* = 39) had the highest Net Total score (M = 29.46, SD = 23.01), followed by the gambling-disorder group (*n* = 33; M = 16.30, SD = 22.11) and the OUD pre-methadone group (*n* = 30; M = 5.03, SD = 27.82). The three-group ANOVA showed a significant cohort effect, F(2,99) = 8.73, *p* < 0.001, and η^2^ = 0.150. Pairwise Welch tests with Holm correction indicated higher performance in controls than in the gambling-disorder and OUD pre-methadone cohorts. The gambling-disorder versus OUD pre-methadone comparison did not reach statistical significance (Table 2).

**Figure 2:**
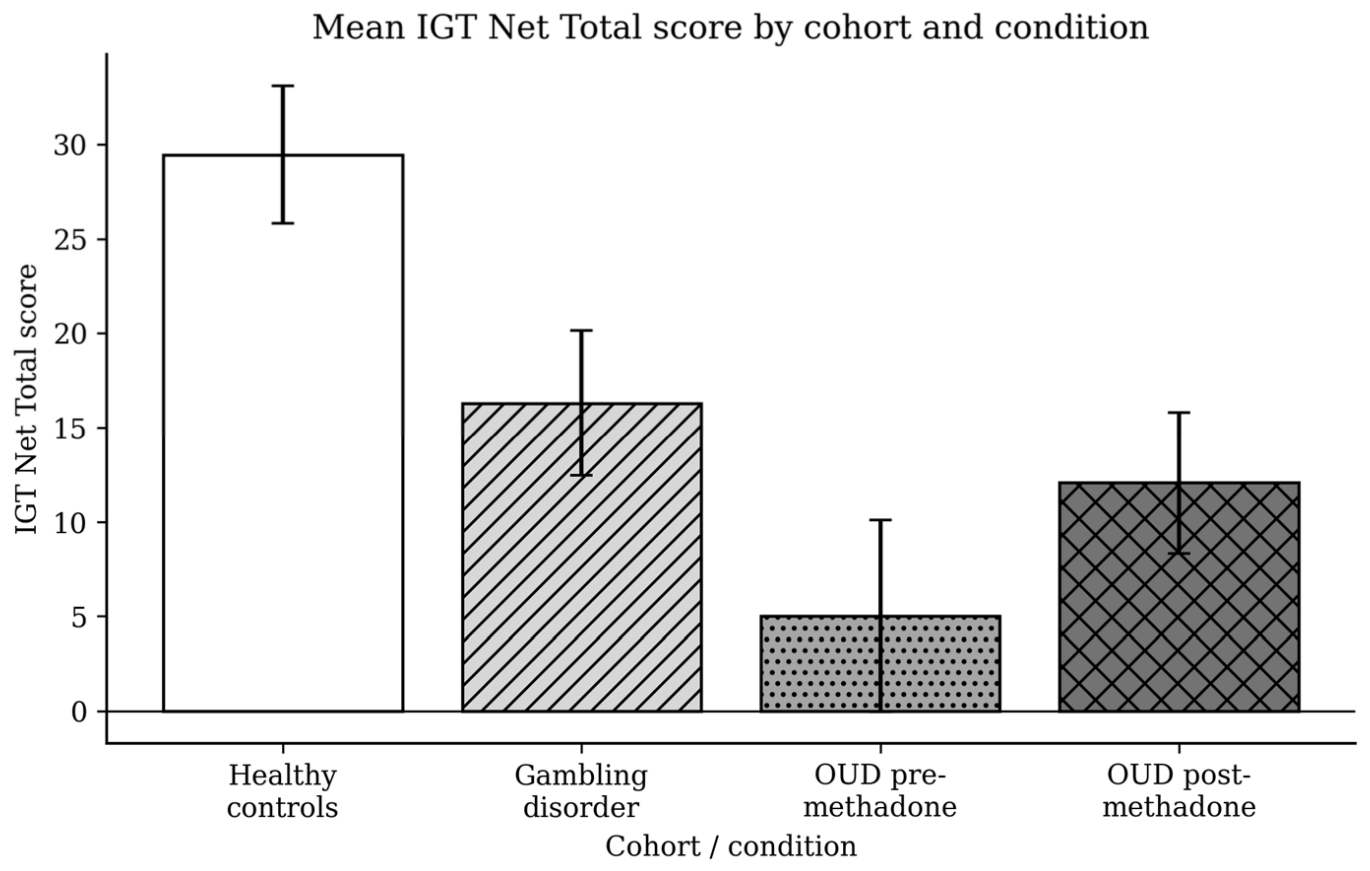
Descriptive mean IGT Net Total scores by cohort and condition. Error bars represent the standard error of the mean (SEM). Outcome denominators are *n* = 39 for healthy controls, *n* = 33 for gambling disorder, *n* = 30 for OUD pre-methadone, and *n* = 27 for OUD post-methadone. The OUD pre- and post-dose bars describe available observations from the same clinical cohort; paired inference was restricted to the 27 complete pairs.

For descriptive cohort-level context, the 30 available pre-dose OUD observations had a mean Net Total of 5.03 (SD = 27.82), whereas the 27 available post-dose observations had a mean of 12.07 (SD = 20.47) (Figure 2). These descriptive means use different denominators and therefore are not themselves a paired mean-change estimate. Inferential comparison was restricted to the 27 participants with complete paired observations. The within-subject condition effect was not statistically significant, F(1,26) = 1.55 and *p* = 0.224. This nonsignificant result does not establish equivalence, and the descriptive post-dose difference should not be interpreted as evidence of acute cognitive restoration.

### 3.2. Block-Wise IGT Trajectories

**Figure 3:**
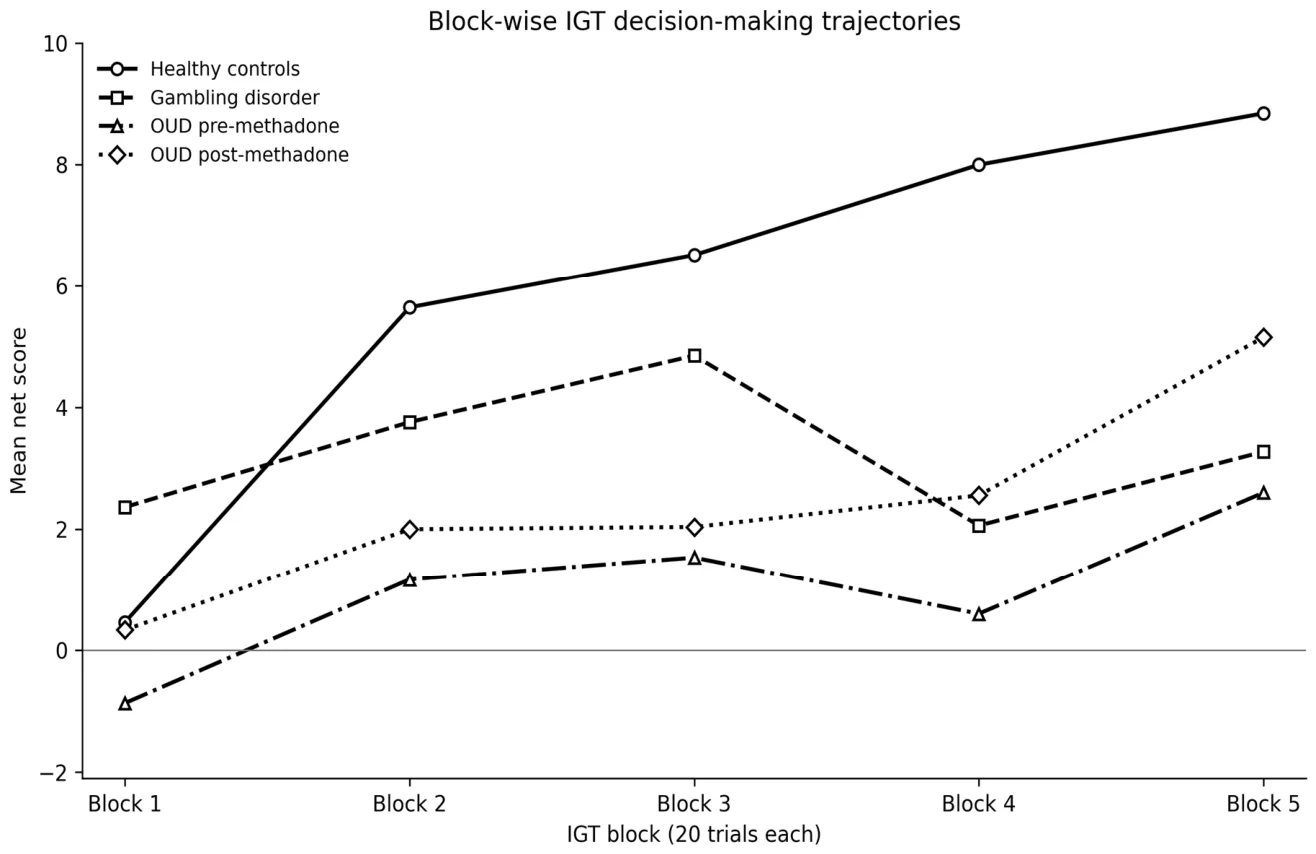
Block-wise IGT decision-making trajectories across five 20-trial blocks. Values are descriptive group means; no block-specific between-cohort inference is implied by this figure.

Block-wise IGT performance was summarized descriptively rather than analyzed with a four-condition between-groups ANOVA, because the OUD pre- and post-methadone observations were obtained from the same participants. Healthy controls showed progressive improvement across the five blocks, with mean net scores of 0.46, 5.64, 6.51, 8.00, and 8.85. In OUD pre-methadone, mean scores remained low across the task (−0.87, 1.17, 1.53, 0.60, and 2.60), whereas the OUD post-methadone trajectory was 0.33, 2.00, 2.04, 2.56, and 5.15. These descriptive profiles are consistent with stronger task learning in controls and more limited learning in OUD, but the pre- versus post-methadone difference cannot be evaluated by treating the two OUD conditions as independent groups (Figure 3).

The gambling-disorder group showed a non-monotonic descriptive profile: 2.36 in Block 1, 3.76 in Block 2, 4.85 in Block 3, 2.06 in Block 4, and 3.27 in Block 5. The observed Block 3-to-4 difference was 2.79 net-score points, followed by partial recovery. The overall within-group block effect previously reported for the gambling cohort was not statistically significant (*p* = 0.35); consequently, the Block 4 decrease should be regarded as an exploratory feature of the group trajectory rather than a statistically established within-group change. The OUD pre- and post-dose block profiles are presented descriptively and no block-specific or condition-by-block inferential conclusion is drawn from these trajectories.

### 3.3. Learning-Curve Interpretation Across Cohorts

The trajectory patterns differed qualitatively across cohorts. Healthy controls showed a descriptive monotonic shift toward advantageous choices, consistent with progressive learning from the task contingencies. The OUD pre-methadone condition remained comparatively flat and low across the task, whereas the OUD post-methadone condition showed a larger descriptive increase in later blocks. However, the within-subject condition effect was not statistically significant, F(1,26) = 1.55 and *p* = 0.224, and the block-wise profiles are descriptive; the apparent post-dose pattern should therefore not be interpreted as evidence that the scheduled methadone dose normalized decision-making.

The gambling disorder trajectory differed from both the monotonic control profile and the relatively flat OUD pre-methadone profile. Performance improved through Block 3, declined in Block 4, and partially recovered in Block 5 (Figure 3). Because the overall block effect within the gambling cohort was not significant, the late decline may reflect true temporal instability, sampling variability, or a combination of both. Its close temporal similarity to the Block 4 decline reported by Lakey et al. [133] makes the observation suitable for targeted replication, but it does not establish a unique pathophysiological phase, cognitive-emotional breakdown, or diagnostic marker. The descriptive block-wise profiles suggest potentially different temporal patterns in this recruited sample. The data are compatible with more stable adaptive learning in healthy controls, persistently low performance in OUD pre-methadone, a descriptive late-task increase in OUD post-methadone, and a non-monotonic trajectory in gambling disorder. These behavioral patterns do not by themselves identify the underlying neural or affective mechanism. Participant-level mixed-effects models, pre-specified block contrasts, and direct measures of executive control, arousal, and loss chasing would be required to determine whether the observed temporal differences are reproducible and mechanistically specific.

## 4. Discussion

In the Iowa Gambling Task (IGT), healthy controls typically exhibit a learning curve characterized by a gradual increase in advantageous choices and higher net scores across successive blocks [134]. In contrast to healthy individuals, impaired decision-making has been reported in both behavioral addictions and OUD. Moreover, the maladaptive patterns of risky behavior differ between individuals with gambling disorder and those with substance-related addictions.

Participants with OUD showed low IGT performance before the scheduled daily methadone dose, with only limited descriptive improvement across blocks. The post-dose assessment showed a numerically higher Net Total and a larger descriptive increase in later blocks than the pre-dose assessment. However, the within-subject condition effect was not statistically significant. The apparent post-dose difference should therefore be interpreted descriptively rather than as evidence that methadone acutely restored or normalized decision-making. Performance also remained below that of healthy controls, consistent with previous reports of persistent decision-making deficits in OUD during maintenance treatment [23,135].

Individuals with gambling disorder also showed impaired IGT performance, but with a more dynamic trajectory than participants with OUD. Performance initially improved through Block 3, followed by a decline in Block 4 and partial recovery in Block 5. Because the overall within-group block effect was not significant, this late decline should be regarded as exploratory rather than evidence of a discrete or reproducible “Net 4” phenomenon. Nevertheless, its timing is consistent with previous observations. Among 221 frequent card players, Lakey et al. [133] reported no initial group differences but subsequently observed poorer advantageous-deck selection in participants with problem and pathological gambling, with a pronounced decline in Block 4. The authors interpreted the return to disadvantageous decks as potentially reflecting loss chasing or greater acceptance of known risk. Thus, our finding is better considered an extension of a previously described late-task instability than a novel block-specific marker (Table 3).

This interpretation is supported, although not specifically for Block 4, by evidence of more frequent episodic chasing and poorer decision-making strategies in individuals with gambling disorder [139]. Such episodes occurred across the task, arguing against a uniquely pathological role for Block 4 while supporting recurrent shifts towards disadvantageous choices as a plausible behavioural mechanism. Later IGT performance may also place greater demands on executive control once deck contingencies have been learned: executive-function measures are more strongly associated with later than early task performance [137], consistent with increasing recruitment of deliberative or “cool” processes alongside affective decision-making [121]. A late decline could therefore reflect difficulty maintaining an advantageous strategy rather than failure to learn task contingencies, although this interpretation remains speculative without direct measures of strategy knowledge, executive control, arousal, or loss chasing.

Heterogeneity within gambling disorder further cautions against a unitary interpretation. IGT impairment and deficient feedback processing have been reported previously, with greater deficits in some gambling subgroups [136], while impaired late-stage decision-making cannot be explained solely by deficient response inhibition [138]. Collectively, these findings suggest that impaired IGT performance in gambling disorder probably reflects interacting disturbances in reward valuation, feedback processing, metacognitive appraisal, and affective and executive control [121].

The conventional Net Total combines selections from A and B and from C and D, but these decks differ not only in long-term expected payoff but also in loss frequency. In particular, the prominent-deck-B phenomenon discussed by Lin et al. [140] indicates that identical aggregate net scores need not imply identical choice strategies. Our data therefore describe overall and block-wise selections rather than identifying a unique executive or affective mechanism. The ecological validity of laboratory decision tasks likewise depends on the outcome of interest; evidence from two nonclinical samples [141] cannot substitute for prospective validation of relapse, real-life loss chasing, or individual prognosis in the present clinical cohorts.

This pattern draws attention to the temporal dimension of decision-making impairment in behavioral addiction. The observed behavioral patterns are consistent with current neurobiological models explaining decision-making mechanisms in individuals with addiction [95,113]. Neurocognitive models relate successful IGT performance to PFC functioning—particularly ventromedial and orbitofrontal regions that integrate reward and punishment history to guide future choices [142,143,144]. Healthy individuals tend to delay receiving large immediate rewards by evaluating long-term benefits—this type of behavioral strategy is attributed to efficient somatic ‘signals from within’ originating in the ventromedial prefrontal cortex and its connections with the limbic system (e.g., the amygdala, the insula) [145,146,147]. In some individuals with addictions, a stronger weighting of immediate over cumulative outcomes is observed or proposed. Both substance-dependent individuals and those with gambling disorder tend to choose options that provide immediate gratification, despite long-term negative consequences—a pattern also reported in individuals with ventromedial prefrontal cortex damage [148]. Previous work has shown that some individuals with substance use disorders perform on the IGT within the range observed in patients with ventromedial prefrontal damage, supporting overlap in decision-making deficits [149]. The term ‘myopia for the future’ describes a disproportionate weighting of immediate over longer-term outcomes in influential accounts of addictive decision-making [148]. Some decision-making profiles in gambling disorder have been discussed in terms of ‘myopia for the future,’ including preference for immediate rewards despite cumulative losses [148]. Compared to healthy individuals, people with gambling disorder exhibit poorer decision-making in the IGT, reflecting an excessive preference for decks with high rewards and high penalties [121]. Neurocognitive models suggest that the tendency to prioritize immediate rewards while ignoring adverse consequences may stem from hyperactivation within the brain’s reward circuitry [150,151,152]. Neurobiological models of addiction emphasize dysregulation of dopaminergic reward circuits, which may contribute to impaired decision-making under uncertainty [153]. Alterations in dopaminergic signaling have been proposed as a key mechanism linking reward processing with addictive behaviors [153].

Models attempt to explain the lack of reward delay by suggesting that the ventral striatum (nucleus accumbens) and associated dopaminergic pathways are hyper-responsive to cues of potential wins in individuals with gambling disorder, even when those wins are accompanied by greater losses [154,155,156,157]. The reward system, being overly sensitive to stimuli associated with immediate gratification, may lead to impulsive decisions without anticipating future negative consequences—a pattern characteristic of individuals with addiction [56,86,99,158]. Models of addiction propose that altered interactions between prefrontal and subcortical dopaminergic structures may favor immediate rewards over longer-term consequences [56,95,158,159,160,161]. Neuroimaging studies conducted on individuals with gambling disorder suggest hyperactivity of the striatum in response to rewards and reduced prefrontal cortex functioning, supporting the triadic neurocognitive hypothesis [121], which posits that an overactive impulsive reward system overrides a weakened reflective system responsible for cognitive control. Similar patterns have also been observed in substance use disorders [56]. Similarly, in the case of substance addictions, chronic drug use is associated with a dysregulation of the fronto-striatal loops: an overactive ‘Go’ system, driven by subcortical reward-related structures such as the nucleus accumbens, amygdala, and ventral tegmental area, and a weakened ‘Stop’ system located in the prefrontal cortex [56,99,158]. This is supported by evidence that addicted individuals show functional and structural deficits in prefrontal regions (including orbitofrontal and anterior cingulate cortex) as well as altered neurotransmission in reward pathways [148,162].

Such theoretical accounts provide a framework for interpreting the lower IGT scores in the OUD cohort, but the present behavioral data do not establish a shift in control to subcortical structures or directly measure PFC regulation. Maintenance treatment with methadone has been discussed as a means of stabilizing aspects of opioid-related reward processing [163]. Methadone, as an opioid receptor agonist, binds to opioid receptors in a manner that stabilizes the functioning of the brain’s reward system, thereby counteracting the neurochemical fluctuations typically associated with heroin use and reducing the intensity of both euphoric and dysphoric states [164]. Methadone may reduce drug craving and stress levels, attenuate autonomic nervous system arousal, and thereby facilitate more effective engagement of cognitive control circuits during decision-making [164,165,166,167]. The numerical post-dose increase observed here may be compared with previous reports, although the present paired condition effect was not significant [135]. Earlier investigations reported reductions in risk-related behavior after a therapeutic dose of methadone and higher net IGT scores in the studied samples [14,168].

Individuals with OUD who are undergoing substitution treatment with methadone perform better on the IGT and experience lower levels of cue-induced drug craving compared to those who have recently undergone detoxification [23,135,169,170]. Neurobiologically, improved performance on the IGT in individuals with OUD following methadone administration may reflect reduced dysregulation of the orbitofrontal cortex and the mesolimbic dopaminergic system, as stabilization of the opioid system attenuates reward circuit hyperactivity and restores prefrontal control [171,172]. Individuals with OUD receiving methadone treatment do not reach the level of IGT performance observed in healthy controls, which may indicate persistent functional deficits within the prefrontal cortex and/or heightened sensitivity of the reward system. Achieving meaningful improvement in risk-related decision-making may require more sustained interventions and a more comprehensive pharmacological treatment approach [173,174]. In the present study, a descriptive decline of 2.79 net-score points was observed from Block 3 to Block 4 in gambling disorder; the overall within-group block effect was not statistically significant, and no confirmatory Block 3-to-4 test was established. One hypothesis is reduced stability of an initially advantageous strategy, but this mechanism was not directly tested.

One possible interpretation of the late decline in decision-making efficiency among individuals with gambling disorder is reduced stability of learned choice strategies over time. Early in the task, partially preserved executive control and sensitivity to losses may support advantageous learning, whereas continued exposure to intermittent high rewards could increase the salience of disadvantageous options. This account is compatible with incentive-salience and loss-chasing models, but it remains speculative because the present study did not measure craving, arousal, subjective strategy, or neural activity during the relevant trial window. The Block 4 pattern should therefore not be equated with a real-world ‘winning streak’ or compulsive gambling episode. Rather, it may serve as a behavioral hypothesis for future studies examining whether prolonged reward exposure alters the balance between learned long-term value and the salience of immediate reward in gambling disorder.

The OUD cohort performed poorly before the scheduled daily methadone dose and showed a numerically higher Net Total score after dosing. This direction is compatible with earlier reports of improved risk-related behavior after methadone administration and with studies in which opioid-maintained patients differed from early-abstinent users [14,135]. Nevertheless, the present paired analysis did not demonstrate a significant main effect of assessment condition. It would therefore be inappropriate to conclude that a single methadone dose “restored” cognitive control based on these data. The fixed pre-dose/post-dose sequence also creates an important alternative explanation: the post-dose session occurred after prior exposure to the same task. Practice, memory for deck contingencies, elapsed time, fatigue, and changes in physiological state may all contribute to the numerical difference. A future study should use an alternative or counterbalanced IGT version, a comparison condition, or a design that separates pharmacological effects from repeated-test learning. The block-wise OUD trajectories are descriptive and are not used to support a specific learning mechanism.

The contrasting trajectories are compatible with, but do not directly demonstrate, different disturbances within cortico-striatal decision systems. Addiction models emphasize impaired integration of long-term outcomes, altered salience of immediate reward, and weakened top–down regulation from prefrontal regions over striatal and limbic systems [56,87,95]. In gambling disorder, neuroimaging studies have demonstrated altered reward-related frontostriatal responses and dopaminergic signaling during monetary decision-making [104,154,175]. Such evidence provides a plausible framework for persistent risk preference, but the present behavioral data cannot specify the underlying neural mechanism.

A theoretical implication of the observed late-task decline is that models of gambling disorder may benefit from considering not only average decision-making performance but also its stability over time. The present pattern raises the hypothesis that sustained exposure to reward-related choices can challenge the maintenance of an advantageous strategy in some individuals with gambling disorder. Such an effect could reflect changes in reward salience, executive control, fatigue, or other task-related processes; the current behavioral data cannot distinguish among these mechanisms. This interpretation is broadly compatible with incentive-salience accounts, which propose that reward-related cues can acquire disproportionate motivational value and interfere with goal-directed control [176], but direct tests using concurrent measures of craving, arousal, and executive function are required.

The clinical relevance of late-task instability remains to be established. If the descriptive Block 4 pattern is replicated prospectively and related to clinically meaningful outcomes, it could motivate studies of interventions designed to support decision stability during prolonged exposure to gambling cues. Candidate approaches might include behavioral strategies that interrupt extended gambling episodes, strengthen recognition of escalating risk, or reduce loss-chasing tendencies. At present, however, the current IGT finding is not sufficient to define a treatment threshold, identify the point at which an individual’s rational decision-making ‘breaks down’, or justify a specific pharmacological or neuromodulatory intervention. Future clinical studies should test whether temporal IGT features add predictive value beyond established measures of gambling severity, impulsivity, and relapse risk.

Future studies should replicate the obtained results and determine whether the dynamics of performance decline during task execution are related to clinical indicators such as gambling severity, impulsivity, or relapse rates. The present findings are broadly consistent with previous research using the IGT, both in the context of substance-related and behavioral addictions, while also pointing to some nuanced differences. Across the literature, individuals with addiction generally perform worse on the IGT compared to healthy controls, a pattern that is well-documented in the literature [14,121,149]. Individuals with gambling disorder, like those with substance addictions, struggle to consistently choose advantageous card decks, which suggests difficulties in learning from negative consequences and resisting stimuli associated with high rewards [121]. Previous studies of gambling disorder suggest that gamblers tend to achieve lower net scores on the IGT compared to healthy individuals, and show a more prolonged tendency to make disadvantageous choices. This has been linked to reward-driven dominance and ‘myopia for the future,’ similar to patterns observed in substance use disorders [107,121,175]. Other research indicates that individuals with gambling disorder may continue to select disadvantageous decks despite accumulating losses, reflecting the persistence of gambling behavior in real life—even in the face of financial and personal harm [177]. These previously reported observations [177] are largely consistent with the behavioral patterns identified in the original research presented here, involving individuals with gambling disorder at the final stage of the IGT. Among individuals with substance use disorders, impaired performance on the IGT has been documented across various substances, including stimulants, alcohol, opioids, and others [95,178]. Individuals with chronic substance use disorders often make decisions resembling those of patients with orbitofrontal cortex damage—they opt for immediate rewards while showing insensitivity to future losses [148]. Incentive sensitization models propose that repeated exposure to reward cues enhances motivational salience and contributes to maladaptive gambling behavior [176].

Previous work has shown that a substantial subset of substance-dependent individuals perform within the range of ventromedial prefrontal patients on the IGT [149]. In the original research presented, individuals with OUD before methadone administration (OUD pre-methadone) demonstrated lower final scores on the IGT compared to healthy controls. This poorer performance on the IGT by individuals with OUD assessed before the scheduled daily methadone dose is consistent with previous reports, further confirming that OUD—like other forms of substance dependence—is associated with significant decision-making deficits [49,179,180]. There is some evidence, however, that different addictions might produce somewhat different IGT profiles [181]. Some studies report that individuals with stimulant use disorders, as well as some individuals with frontal lobe damage, often make disadvantageous decisions from the outset and show no improvement in decision-making over time [96,182,183]. Findings from studies on individuals with gambling disorder suggest a temporally limited learning potential, which prevents them from achieving the level of cognitive performance observed in healthy individuals [184]. Gambling disorder shares several clinical and neurocognitive features with substance use disorders, including altered reward processing and impaired executive control [185]. Because gambling disorder does not involve direct exposure to an exogenous neurotoxic substance, differences from substance use disorders may partly reflect distinct neurobiological and cognitive profiles [186]. This distinction may help explain why participants with gambling disorder showed relatively better early IGT performance than participants with OUD in the present sample, although the current behavioral data cannot establish the neural basis of that difference. One hypothesis is that the stability of advantageous strategy use may be more limited in gambling disorder during prolonged task performance. By the end of the task, net scores in the gambling group were closer to those observed in OUD [23]. Another relevant question is whether treatment duration, abstinence, withdrawal, or craving modifies decision-making efficiency. In the present study, IGT performance was numerically higher after methadone administration than before the scheduled daily dose, but the within-subject methadone effect was not statistically significant. Kriegler et al. [135] reported higher IGT performance and lower drug craving in individuals receiving opioid substitution treatment than in participants who had recently undergone heroin detoxification. The prior literature raises the possibility that some decision-making deficits are state-dependent and may vary with withdrawal or craving; the present data, however, do not establish acute pharmacological reversibility. In comparison to individuals with OUD, those with gambling disorder—like other behavioral addictions—lack a direct pharmacological intervention that would straightforwardly improve functioning. As a result, clinical treatment relies primarily on psychosocial interventions [187]. It is noteworthy that even after methadone administration, individuals with OUD do not reach the same level of performance on the IGT as healthy controls. The post-methadone condition was associated with numerically better decision-making performance in individuals with OUD, although the within-subject effect was non-significant and performance remained below that of healthy controls [164,188]. The present behavioral pattern is consistent with the broader literature indicating that both substance and behavioral addictions are associated with dysfunctional decision-making on tasks such as the IGT, stemming from an imbalance between impulsive reward-seeking and regulatory control mechanisms [121]. The present work builds on earlier observations [23] and describes a nonmonotonic task trajectory in gambling disorder, including an exploratory late-task decline. This descriptive finding suggests that the dynamics of decision-making in gambling disorder may differ from those observed in individuals with OUD [14,23]. The observed descriptive difference in temporal decision-making patterns between gambling disorder and OUD warrants consideration in the design of future research. This distinction may inform future mechanistic and clinical research, but its diagnostic or therapeutic value requires prospective validation.

The approximately 90-min post-dose interval was selected in light of our earlier IGT and oculomotor investigations [14,127,131] and preliminary clinical observations of subjectively pronounced post-dose effects. It does not represent an expected peak-plasma-concentration assessment. Cognitive findings in methadone-maintained patients have been reported to differ according to assessment timing and cognitive domain [189]. Separately, test–retest research indicates that repeated IGT administration can change task performance [190], although the participants and interval of that study differ from our same-day clinical procedure. The present fixed order therefore combines dosing state with prior IGT exposure, elapsed time, and potential fatigue; the session contrast cannot isolate an acute pharmacological effect.

Several limitations materially constrain interpretation. First, all participants were men, limiting generalizability to women and mixed-sex clinical populations. Second, recruitment settings differed substantially: the gambling cohort was recruited in a prison, controls through academic institutions, and the OUD cohort from substitution-treatment programmes. Although all participants underwent study-specific clinical qualification by an addiction psychiatrist, group differences may still partly reflect incarceration, education, socioeconomic background, age, treatment engagement, or other cohort-level factors rather than diagnosis alone. The control group was younger on average than the two clinical groups. Third, residual clinical heterogeneity beyond the variables reported in Table 1 should be considered when interpreting between-cohort contrasts. Fourth, the Block 4 decline was identified from the observed trajectory and was not tested with a preregistered within-gambling Block 3 versus Block 4 contrast or a full mixed-effects model; it should therefore be considered hypothesis-generating. Fifth, the pre-/post-methadone comparison was not counterbalanced and may be affected by practice, order, elapsed-time, or fatigue effects. Replication with larger, demographically better-matched samples, explicitly planned repeated-measures analyses, and participant-level mixed-effects modeling is required before the temporal patterns can be considered reliable clinical phenotypes.

## 5. Conclusions

The observed temporal patterns of decision-making in gambling disorder and OUD have potential theoretical and clinical relevance, but they should be interpreted within the limits of the behavioral design. Both groups showed lower IGT performance than healthy controls, although their temporal profiles differed descriptively. These findings are compatible with neurobiological models emphasizing altered interactions between prefrontal control systems and striatal–limbic reward circuitry, but the present study did not measure neural activity and therefore cannot demonstrate prefrontal hypoactivity or subcortical hyperactivity directly. The data instead show that the temporal evolution and stability of disadvantageous choice behavior may differ across addiction types. In gambling disorder, the late-task decline is an exploratory pattern that warrants replication; in OUD, performance was low before the daily methadone dose and numerically higher after dosing, but the within-subject methadone effect was not statistically significant. If future studies confirm reliable temporal markers, they may help refine behavioral phenotyping and the design of interventions aimed at maintaining adaptive decision-making under sustained reward-related demands. At the group level, IGT results showed differences between the recruited control and clinical cohorts, alongside descriptively different temporal patterns; these mean differences do not validate an individual diagnostic test. Overall, the findings support analysis of decision-making as a dynamic process and provide a behavioral framework for future studies linking temporal IGT profiles with executive control, reward processing, loss chasing, and clinically relevant outcomes.

## Figures and Tables

**Table 1:** Sociodemographic and clinical characteristics of the study cohorts.

Characteristic	Category/Criterion	Gambling Disorder (*n* = 33)	Opioid Use Disorder (*n* = 30)	Healthy Controls (Enrolled *n* = 40)
Age, mean ± SD, years		31.64 ± 5.16	29.53 ± 5.20	25.53 ± 5.34
Recruitment setting		Bydgoszcz-Fordon Prison	Substitution programs, Bydgoszcz and Gdańsk	Academic institutions, Kraków and Bydgoszcz
IGT assessment		Single assessment; 33 complete IGT records	30 pre-dose assessments; 27 complete pre-/post-dose pairs	40 enrolled; 39 complete IGT records
Education	Primary	18 (54.5%)	16 (53.3%)	13 (32.5%)
	Secondary	10 (30.3%)	8 (26.7%)	16 (40.0%)
	Higher	5 (15.2%)	3 (10.0%)	11 (27.5%)
	Not recorded	—	3 (10.0%)	—
Methadone dose	Mean ± SD, mg/day	—	70.4 ± 11.9	—

Note. Values are *n* (%) unless otherwise indicated. The cohort columns describe enrolled participants for the reported sociodemographic characteristics. Forty healthy controls were enrolled; one participant discontinued the IGT before completion, leaving 39 complete control outcome records. The OUD cohort comprised 30 baseline participants, of whom 27 completed both pre- and post-dose assessments and were included in paired inference. No missing outcome values were imputed. Percentages use the cohort denominator specified in each column. “Not recorded” denotes entries without recorded category information. SD, standard deviation; IGT, Iowa Gambling Task; OUD, opioid use disorder.

**Table 2:** Independent-cohort comparisons for IGT Net Total.

Comparison	Mean Difference	95% CI	Holm-Adjusted *p*	Hedges g	Interpretation
Control vs. Gambling disorder	13.16	2.53 to 23.79	0.032	0.58	Control higher
Control vs. OUD pre-methadone	24.43	11.86 to 37.00	<0.001	0.96	Control higher
Gambling disorder vs. OUD pre-methadone	11.27	−1.50 to 24.04	0.083	0.45	Not significant

Note: Pairwise comparisons use Welch *t* tests with Holm correction; confidence intervals are unadjusted Welch 95% CIs. Hedges g is reported as a standardized mean difference, positive when the first named cohort has a higher Net Total score.

**Table 3:** Prior IGT findings relevant to the temporal gambling-disorder trajectory observed in the present study.

Study	Sample/Design	Relevant Result	Relevance to Present Study
Lakey et al. (2007) [133]	221 frequent card players; non-problem, problem, and pathological gambling; five IGT blocks	Marked decrease in advantageous choices in Block 4 in problem and especially pathological gamblers; loss chasing proposed as one interpretation	Closest direct precedent for the present Block 4 decrease
Goudriaan et al. (2005) [136]	Pathological gambling vs. alcohol dependence, Tourette syndrome, and controls	PG showed poorer IGT performance and feedback processing; slot-machine gamblers were more impaired than casino/card gamblers	Supports heterogeneity by gambling modality and feedback processing
Brand et al. (2007) [137]	Healthy adults; IGT and executive-function measures	Later IGT blocks were more strongly associated with executive functions and increasingly reflected decision-making under known risk	Provides a cognitive framework for interpreting late-task instability
Kertzman et al. (2011) [138]	Pathological gamblers and controls; early vs. later IGT performance plus inhibition tasks	PG showed impaired decision-making; late-stage IGT impairment was not explained by response-inhibition deficits alone	Argues against a simple inhibition-only explanation
Brevers et al. (2013) [121]	Review of IGT research in gambling disorder	Later IGT stages may involve both affective (“hot”) and deliberative (“cool”) executive processes; gambling deficits show substantial heterogeneity	Supports multi-process interpretation and cautious mechanistic claims
Linnet et al. (2006) [139]	61 pathological gamblers and 39 non-pathological gamblers; modified IGT/Mouse Game; sequence-based chasing index	More episodic chasing and poorer decision-making in pathological gambling; chasing episodes occurred throughout the task rather than only in Block 4	Supports loss chasing as a plausible mechanism, but not a Block 4-specific effect

## Data Availability

The data supporting the findings of this study, including anonymized source IGT/PEBL records and documentation of the batch-processing procedure where permissible, are available from the corresponding author upon reasonable request. Due to the clinical nature of the data and participant privacy considerations, only anonymized data can be made available.
